# Bacterial Valorization of Lignin: Strains, Enzymes, Conversion Pathways, Biosensors, and Perspectives

**DOI:** 10.3389/fbioe.2019.00209

**Published:** 2019-09-03

**Authors:** Siseon Lee, Minsik Kang, Jung-Hoon Bae, Jung-Hoon Sohn, Bong Hyun Sung

**Affiliations:** ^1^Synthetic Biology and Bioengineering Research Center, Korea Research Institute of Bioscience and Biotechnology, Daejeon, South Korea; ^2^Department of Biosystems and Bioengineering, Korea University of Science and Technology, Daejeon, South Korea

**Keywords:** lignin valorization, bacterial lignin degradation, enzymatic depolymerization, bacterial laccase, dye-decolorizing peroxidase, biosensor

## Abstract

Lignin, an aromatic polymer found in plants, has been studied for years in many biological fields. Initially, when biofuel was produced from lignocellulosic biomass, lignin was regarded as waste generated by the biorefinery and had to be removed, because of its inhibitory effects on fermentative bacteria. Although it has since proven to be a natural resource for bio-products with considerable potential, its utilization is confined by its complex structure. Hence, the microbial degradation of lignin has attracted researchers' interest to overcome this problem. From this perspective, the studies have primarily focused on fungal systems, such as extracellular peroxidase and laccase from white- and brown-rot fungi. However, recent reports have suggested that bacteria play an increasing role in breaking down lignin. This paper, therefore, reviews the role of bacteria in lignin and lignin-related research. Several reports on bacterial species in soil that can degrade lignin and their enzymes are included. In addition, a cellulolytic anaerobic bacterium capable of solubilizing lignin and carbohydrate simultaneously has recently been identified, even though the enzyme involved has not been discovered yet. The assimilation of lignin-derived small molecules and their conversion to renewable chemicals by bacteria, such as muconic acid and polyhydroxyalkanoates, including genetic modification to enhance their capability was discussed. This review also covers the indirect use of bacteria for lignin degradation, which is concerned with whole-cell biosensors designed to detect the aromatic chemicals released from lignin transformation.

## Introduction

Lignin was considered a typical industrial by-product, available in pulp and paper waste, agricultural residue, and other hydrolytic industries (Gottlieb and Pelczar, 1951; Reddy and Yang, 2005; Santos et al., 2013). Specifically, large quantities of lignin were produced by biorefineries, as an alternative for industries that manufactured petroleum-based products. However, interest in lignin has dramatically increased in recent years as the demand for biomass-based products increased as a consequence of the energy crisis and the environmental problems humankind is facing.

Lignin is a complex aromatic polymer of high heterogeneity, and is attached to carbohydrate via an ester linkage within a plant to provide cell wall rigidity and protection. It comprises a considerable percentage of lignocellulose (as much as 30%) although it differs according to the type of source (Vanholme et al., 2010). Lignin includes phenolic compounds, of which there are mainly three groups, i.e., guaiacyl (G), syringyl (S), and hydroxyphenyl (H), which are polymerized via cross-linking mediated by an enzymatic radical reaction, giving rise to β-O-4 bonding dominantly and other C-C and C-O bonding (Ralph et al., 2004). Because of the intrinsic recalcitrance of lignin, it was simply regarded as waste to be removed from the pretreated biomass at the beginning of biofuel production, which is hampered by lignin-derived toxicity (Ezeji et al., 2007). To avoid this problem, even until recently, methods to recover the lignin from the pretreated liquid have mainly been reported, or strategies have been suggested to construct robust strains under lignin-derived toxic conditions (Cho et al., 2009; Lu et al., 2013; Lee et al., 2015). Nevertheless, lignin has vast potential to be used in biological systems as the second-most abundant source of aromatic compounds on the Earth. Thus, a process with a new perspective is required to not only decrease the recalcitrance of biomass but also enable lignin valorization (Ragauskas et al., 2014).

The first step in biological lignin valorization is the decomposition of the lignin polymer. The natural depolymerization of lignin by white- or brown-rot fungi, which produce extracellular oxidative enzymes, including laccase and peroxidases, has been intensively studied (Dashtban et al., 2010). Laccase can oxidize phenolic units with oxygen as an electron acceptor. The laccase from *Trametes versicolor* was shown to degrade the β-O-4 link within a non-phenolic lignin model (Kawai et al., 2002). In addition, treatment using the laccase within *T. villosa* removed lignin from wood (Gutiérrez et al., 2012). Other enzymes reactive to lignin are heme-containing peroxidases, such as lignin peroxidase (LiP), manganese peroxidase (MnP), and versatile peroxidase (VP), which needs hydrogen peroxide or a metal mediator. *Phanerochaete chrysosporium* has been found to be able to secrete several oxidases, and, among them, LiP has been used for a comparative analysis of other novel enzymes because of their high activity to lignin (Singh and Chen, 2008). In spite of the high catalytic efficiency of fungal enzymes, the challenges of expression are obstacles for their use in large-scale lignin treatment. In contrast, the lignin depolymerization activity of bacterial enzymes has been reported for limited cases. However, several species of bacteria capable of depolymerizing lignin have been isolated with lignin-modifying enzymes (Brown and Chang, 2014). Even more, the bacteria have a versatile metabolic pathway where lignin-derived compounds can be transformed into high-value chemicals (Jiménez et al., 2002). Reflecting this, the synthesis of polyhydroxyl-alkanoates (PHAs), for use as bioplastics, has been studied within soil bacteria, such as *Pseudomas putida*, using lignin-derived aromatic compounds (Linger et al., 2014).

In this review, therefore, we present research related to lignin valorization, but remain deeply focused on bacterial activity, which is covered in two sections, the first on lignin depolymerization and the second on the conversion of lignin to value-added products. It should be noted that the concept herein is to distinguish between lignin decomposition for the breakdown of polymers, and the degradation of lignin for the catabolism of lignin-derived phenolics. This includes the use of bacteria as an alternative approach to improve lignin valorization, such as the use of a bacterial sensor to screen enzymes and strains with exceptional catalytic properties.

## Lignin Depolymerization to Produce Low-Molecular-Weight Phenolic Compounds

Lignin valorization by bacteria first requires lignin to be depolymerized to low-molecular-weight (LMW) phenolic compounds. Bacteria and enzymes in the bacterial strains capable of modifying lignin have been reported.

### Bacterial Species Capable of Lignin Decomposition

Lignolytic microbes have been identified for years from various habitats, suggesting that they are widely distributed throughout nature, such as in soil, wood, plants, and even the gut. Among them the breakdown of lignin by bacterial strains has not been studied as widely as fungi, but several strains within the class of actinomycetes and proteobacteria are known. In general, *Sterptomyces, Rhodococcus, Pseudomonas*, and *Bacillus* strains have been reported to have lignin decomposition ability. These results are summarized in Table 1 and the details are presented below.

**Table 1:** Lignin modifying bacterial strains.

**Genus**	**Species**	**Substrates**	**Evidences**	**References**
Streptomyces	*Streptomyces viridosporus* T7A	Corn-stover lignocellulose	Lignin-derived APPL formation	Crawford et al., 1983
		Wheat straw biomass	Increased carbohydrate/lignin ratio	Zeng et al., 2013
	*Streptomyces badius* ATCC 39117	^14^C-labeled plant lignin	Detection of ^14^CO_2_	McCarthy, 1987
	*Streptomyces coelicolor* A3(2)	Grass lignocellulose	Lignin-derived APPL formation	Majumdar et al., 2014
	*Amycolatopsis* sp. 75iv2	Soft and hard wood	Weight loss in lignin	Antai and Crawford, 1981
		Grass lignocellulose	Lignin-derived APPL formation	Brown et al., 2011
Rhodococcus	*Rhodococcus jostii* RHA1	Fluorescent lignin	Increase of fluorescence	Ahmad et al., 2010
		Kraft lignin	Sole carbon source for growth	Mycroft et al., 2015
	*Rhodococcus erythropolis*	Nitrated wheat lignin	Increase of absorbance at 430 nm	Taylor et al., 2012
	*Rhodococcus opacus* DSM 1069	Organosolv lignin	Sole carbon source for growth	Kosa and Ragauskas, 2013
	*Rhodococcus opacus* PD630	Organosolv lignin and corn stover lignin	Sole carbon source for growth	Kosa and Ragauskas, 2013; He et al., 2017
Enterobacter	*Enterobacter lignolyticus* SCF1	Kraft lignin	Increased growth with lignin addition	DeAngelis et al., 2011
			Up-regulation of genes involved in lignin depolymerization	DeAngelis et al., 2013
	*Enterobacter aerogenes*	Kraft lignin	Sole carbon source for growth	Deschamps et al., 1980
	*Enterobacter soil* sp. nov.	Kraft lignin	Sole carbon source for growth	Manter et al., 2011
Pseudomonas	*Pseudomonas putida* KT2440	Alkali-pretreated liquor	Sole carbon source for growth	Salvachúa et al., 2015
	*Pseudomonas putida* A514	Alkali insoluble lignin	Sole carbon source for growth	Lin et al., 2016
	*Pseudomonas putida* NX-1	Kraft lignin	Reduced particle size and guaiacyl unit decrease	Xu et al., 2018
Bacillus	*Bacillus* sp.	Kraft lignin	Sole carbon source for growth	Bandounas et al., 2011
	*Bacillus pumilus*	Kraft lignin	Decrease of molecular weight	Huang et al., 2013
	*Bacillus atrophaeus*	Kraft lignin	Decrease of molecular weight	Huang et al., 2013
	*Bacillus sp*. ITRC-S8	Kraft lignin	Growth and decolorization	Raj et al., 2007
	*Bacillus ligninphilus*	Alkaline lignin	Release of monomeric aromatic compounds	Zhu et al., 2017
	*Paenibacillus glucanolyticus*	BioChoice lignin	Decreased average molecular weight of lignin	Mathews et al., 2016
Others	*Thermobifida fusca*	Switch grass and corn stover	Sole carbon source for growth^a^	Deng and Fong, 2011
	*Clostridium thermocellum*	Populus lignin	Decreased β-O-4 linkage and increased S/G index	Akinosho et al., 2017
	*Caldicellulosiruptor bescii*	Switch grass	Release of lignin-derived aromatic compounds	Kataeva et al., 2013

Several *Streptomyces* strains are well-known for their ability to decompose lignin. *Streptomyces viridosporus* T7A is one of the best-studied strains, as a type of actinobacteria, resembling fungi in filamentous form and producing extracellular lignin-degrading enzymes. Crawford et al. (1983) reported that the intermediate formed from the culture of *S. viridosporus* T7A on corn stover lignocellulose was identified as a lignin-derived acid-precipitable polymeric lignin (APPL), enriched with phenolic hydroxyl groups. Recently, the bio-decomposition of lignin was demonstrated by more intensive chemical compositional analysis including Fourier-transform infrared spectroscopy (FT-IR), pyrolysis gas chromatography–mass spectrometry (Py-GCMS), and ^13^C cross polarization/magic angle spinning nuclear magnetic resonance (CP-MAS NMR) spectroscopy. The results revealed an increased carbohydrate/lignin ratio, and the deduction of the guaiacyl unit in lignin after *S. viridosporus* T7A cultivation on wheat straw biomass (Zeng et al., 2013). Other strains in this *Streptomyces* genus also showed activity toward lignin decomposition; for example, *S. badius* ATCC 39117 decomposed ^14^C-labeled plant lignin (McCarthy, 1987) and *S. coelicolor* A3(2) generated APPL in a medium with grass lignocellulose with the help of small laccase (Majumdar et al., 2014). *Amycolatopsis* sp. 75iv2, formerly *S. setonii* and *S. griseus* 75iv2, was grown on softwood, hardwood, and grass lignocellulose for 12 weeks, finally resulting in 34, 29, and 39% weight loss in lignin, respectively, which was comparable with the weight loss by *S. viridosporus* T7A (Antai and Crawford, 1981). The production of APPL, as shown in *Streptomyces*, was also reported for *Amycolatopsis* sp. 75iv2 on grass lignocellulose (Brown et al., 2011)

*Rhodoccocus* species are also an attractive strain for lignin breakdown. *R. jostii* RHA1, a soil bacterium, was initially identified as a polychlorinated biphenyl (PCB) degrader (Seto et al., 1995). Then, Ahmad et al. (2010) discovered its lignin-degrading activity by an assay using fluorescently modified lignin, and reported that it directly utilized kraft lignin and wheat straw lignocellulose as the sole carbon source within the medium to generate bio-products such as aromatic dicarboxylic acids and vanillin (Sainsbury et al., 2013; Mycroft et al., 2015). *R. erythropolis*, another PCB-degrading bacterium isolated from termite gut (Chung et al., 1994), also exhibited lignin metabolizing activity, which was detected by UV-vis assay using nitrated-lignin from wheat (Taylor et al., 2012). Moreover, the colony forming unit (CFU) of two oleaginous *R. opacus* DSM 1069 and PD630 on ethanol organosolv lignin as a sole carbon and energy source was increased over 300 times compared to the inoculum amount by 7 days, suggesting their capability of decomposing lignin (Kosa and Ragauskas, 2013). Although growth is slower than that of *R. jostii* RHA1, *R. opacus* PD630 was also reported to grow with alkali corn stover lignin as the only substrate (He et al., 2017).

Distinctive types of lignolytic bacteria, which are able to solubilize carbohydrate and lignin within lignocellulose simultaneously, are known. One example is *Thermobifica fusca*, which is an aerobic thermophile that harbors both enzymes in charge of lignin modification and cellulose hydrolysis (Wilson, 2004; Rahmanpour et al., 2016). This strain, with a heterologous expression of alcohol dehydrogenase, produced 1-propanol from untreated switch grass and corn stover biomass, implying that lignin was decomposed to enable cellulase to gain sufficient access to generate the carbon source (Deng and Fong, 2011). *Clostridium thermocellum*, which is an anaerobic and thermophilic organism, also have a similar function. Akinosho et al. (2017) characterized the plant cell wall after treatment with *C. thermocellum* and suggested that the β-*O*-4 linkage, which is dominantly found in lignin, was decreased and the syringyl/guaiacyl (S/G) index was increased by this strain. *Caldicellulosiruptor bescii*, originally isolated from a geothermally heated pool, has also attracted researchers' interest owing to its cellulytic and lignolytic activity. This bacteria was successfully grown on untreated switchgrass biomass at 78°C and released a number of lignin-derived aromatic compounds in the culture medium (Kataeva et al., 2013). Furthermore, engineered *C. bescii*, as *T. fusca*, utilized untreated biomass for ethanol production (Chung et al., 2014). These strains are noteworthy as strong lignin decomposers although they use sugars liberated from the biomass, rather than lignin, as their actual energy source.

DeAngelis et al. (2011) isolated a facultative anaerobic organism, *Enterobacter lignolyticus* SCF1, from rainforest soil by using a medium containing alkali-treated lignin as the sole carbon source. *E. lignolyticus* SCF1 was grown anaerobically on xylose, showing higher cell density by more than 2-fold with the addition of lignin, which infers the strain is capable of depolymerizing lignin. This was proven again in their succeeding research by transcriptomic and proteomic analyses, showing the up-regulation of four enzymes involved in the lignin depolymerization, such as dye-decolorizing peroxidase (DyP)-type peroxidase and glutathione S-transferase, in kraft lignin-amended sample (DeAngelis et al., 2013). Other strains *E. aerogenes* and *E. soil* sp. nov. were also isolated from soil using a culture medium containing kraft lignin as the sole carbon source. *E. aerogenes* is able to assimilate lignin and lignin-derived aromatics (Deschamps et al., 1980; Manter et al., 2011).

*Pseudomonas* strains are also attractive for use in lignin depolymerization with high potential for application in industrial biotechnology (Poblete-Castro et al., 2012; Nikel and de Lorenzo, 2018). Lignin decomposition by *Pseudomonas* species was first tested using a lake isolate by detecting the release of CO_2_ from ^14^C-labeled lignin (Haider et al., 1978). Several decades later, the results of many studies on lignolytic *Pseudomonas* strains were published, in which *P. putida* has been studied as an important example. The capability of *P. putida* KT2440 to depolymerize the lignin was indirectly determined by analyzing *mcl*-PHA produced from alkali-pretreated liquor (APL) with supplementation of ^13^C-labeled compound (Linger et al., 2014). *P. putida* KT2440, as well as its parental strain harboring the mega-plasmid, *P. putida* mt-2, was shown to decrease a significant amount of high molecular weight (HMW) component in APL by nearly 30% after seven-day culture, as measured by Klason lignin analysis and gel permeation chromatography (GPC) analysis (Salvachúa et al., 2015). Another *P. putida* A514, selected from among 15 *P. putida* strains for carbon source utilization, exhibited sufficient growth on the minimal medium supplemented with 1% alkali-insoluble lignin (Lin et al., 2016). A novel isolate, designated as *P. putida* NX-1, was also found to be capable of lignin modification (Xu et al., 2018). After the treatment of lignin with this strain, the morphology and chemical bonds were observed to change by using scanning electron microscopy (SEM) and FT-IR analysis, respectively, resulting in reduced particle size and decrease in the guaiacyl unit. In the case of *P. fluorescens*, although its lignolytic potential was reported with the identification and characterization of extracellular enzyme involved in the lignin deconstruction (Rahmanpour and Bugg, 2015), the actual decomposition of lignin has not been reported yet.

Many strains belonging to *Bacillus* genus are able to decompose lignin polymer. Especially, several isolates from soil, sediment, and sludge have recently been found and characterized for lignin treatment. A soil bacterium screened using kraft lignin was identified as *Bacillus* sp., showing 99% similarity to *B. cereus* and *B. thuringiensis* by 16S rRNA genotyping. This bacterium showed increased CFU after 72 h of culture including the HMW fraction of kraft lignin as the sole carbon source (Bandounas et al., 2011). Huang et al. (2013) also isolated several bacteria from rainforest soil in Peru, two of which, *B. pumilus* and *B. atrophaeus*, were chosen based on their high lignin-degrading activity. The GPC of kraft lignin products degraded by these two strains showed 50–70% removal of large lignin fragments that were extracted by dioxane. In the following evaluation using poplar biomass, a number of compounds were detected by LC-MS analysis, implying that lignin modification occurred to give LMW compounds. One isolate, ITRC-S8, from a sludge sample collected from the pulp industry showed efficient growth on kraft lignin and decolorization of the culture over time, and the reduction of color was well-correlated with that of kraft lignin within the medium (Raj et al., 2007). Zhu et al. (2017) published results, not only relating to the decolorization of the culture medium containing alkaline lignin but also concerning the formation of several compounds with a single phenol ring by *B. ligniniphilus* L1. In addition, *Paenibacillus* strains, formerly in the class of *Bacillus*, were studied to investigate the degradation of polymeric lignin (Chandra et al., 2008; Mathews et al., 2016). GPC analysis of *Paenibacillus glucanolyticus*, which was examined with various substrates including BioChoice lignin under anaerobic or aerobic conditions, showed that the average molecular weight of BioChoice lignin in the culture supernatant had decreased (Mathews et al., 2016).

Lignin deconstruction by *Citrobacter* and *Klebsiella* genus has been proposed, and a culture consisting of a mixture of these two was reported to improve the efficiency. Salvachúa et al. (2015) reported that *C. freundii* did not exhibit efficient bacterial growth on APL and lignin conversion in comparison with other strains examined, including *P. putida* KT2440. However, when *C. freundii* was co-cultured with another *Citrobacter* sp. isolated from sludge sample, effective decolorization of kraft lignin as much as 62% was found (Chandra and Bharagava, 2013). Moreover, high-performance liquid chromatography (HPLC) and GC-MS analyses indicated the formation of new metabolites, such as tri-, tetra-, and penta-chloro phenols. *K. pneumoniae* NX-1 was shown to be able to modify lignin with a 23.8% reduction in absorbance at 280 nm (A_280_) (Xu et al., 2018). In addition, the mixed culture of *K. pneumonia* and *B. subtilis* that was isolated from the sludge, was more efficient in terms of growth and lignin reduction (Yadav and Chandra, 2015).

Apart from this, bacterial lignin depolymerization was reported in *Novosphingobium, Cupriavidus basilensis, Norcadia, Pandoraea* species, among others (Haider et al., 1978; Chen et al., 2012; Shi et al., 2013; Si et al., 2018). Further, *Pandoraea* strains were used to assist with the pretreatment of biomass by facilitating lignin depolymerization by their lignolytic ability (Kumar et al., 2016; Liu et al., 2018).

### Enzymes Involved in Lignin Depolymerization

In general, bacteria that grow in lignin secrete various oxidative enzymes that assist in lignin depolymerization or modification. Peroxidases such as fungal lignolytic peroxidases were rarely found in the bacteria. DyP-type peroxidases and laccases play an important role in bacterial lignin depolymerization (de Gonzalo et al., 2016). These are summarized in Table 2 and demonstrated below in detail.

**Table 2:** Enzymes involved in lignin depolymerization.

**Enzyme**	**Protein**	**Origins**	**Substrates**			**References**
			**Monomeric lignin^a^**	**Dimeric lignin^b^**	**Polymeric lignin^b^**	
**Dye-decolorizing peroxidase**						
*Subfamily A*	DyP	*Bacillus subtilis* KCTC2023	ABTS	VGE	–	Min et al., 2015
		*Thermobifida fusca*	ABTS	GGE	Kraft lignin	Rahmanpour et al., 2016
		*Saccharomonospora viridis*	2,6-DMP	–	–	Yu et al., 2014
*Subfamily B*	DyPB	*Rhodococcus jostii* RHA1	ABTS	GGE	Nitrated lignin and kraft lignin	Ahmad et al., 2011
	DyP1B	*Pseudomonas fluorescens* Pf-5	Guaiacol	–	Kraft lignin and wheat straw cellulose	Rahmanpour and Bugg, 2015
	DyP	*Pseudomonas putida* MET94	Syringaldehyde	GGE	Kraft lignin	Brissos et al., 2017
		*Klebsiella pneumoniaea*	ABTS	–	–	Pfanzagl et al., 2018
		*Enterobacter lignolyticus*	ABTS	–	–	Shrestha et al., 2017
*Subfamily C*	DyP2	*Amycolaptosis* sp. 75iv2	ABTS	GGE	–	Brown et al., 2012
		*Streptomyces avermitilis*	ABTS	–	–	Sugawara et al., 2017
	AnaPX	*Anabaena* sp. strain PCC7120	Syringaldehyde	–	–	Ogola et al., 2009
**Laccase**						
*Laccase*	Lac	*Streptomyces coelicolor* A3(2)	ABTS	GGE and VGE	Ethanosolv lignin	Majumdar et al., 2014
		*Streptomyces lividans* TK24	ABTS	GGE and VGE	Ethanosolv lignin	
		*Streptomyces vifidosporus* T7A	ABTS	GGE and VGE	Ethanosolv lignin	
		*Amycolaptosis* sp. 75iv2	ABTS	GGE and VGE	Ethanosolv lignin	
	Lac4	*Pantoea ananatis* Sd-1	Guaiacol	–	lignin	Shi et al., 2015
	SilA	*Streptomyces ipomoea*	Sinapic acid	–	Kraft lignin	Moya et al., 2011
*Multi-copper oxidase (Laccase-like multicopper oxidase, LMCO)*	CueO	*Escherichia coli*	2,6-DMP	–	PAH^c^	Grass and Rensing, 2001; Zeng et al., 2011
		*Ochrobactrum* sp.	ABTS	GGE and DDVA	Ca-lignosulfonate	Granja-Travez et al., 2018
	CotA	*Bacillus subtilis*	Sinapic acid	–	–	Ihssen et al., 2015
		*Bacillus licheniformis*	Sinapic acid	–	–	Koschorreck et al., 2008
		*Bacillus pumilus*	Guaiacol	–	–	Ihssen et al., 2017
	CopA	*Pseudomonas stutzeri*	ABTS	–	HP-L	Strachan et al., 2014
		*Pseudomonas putida* KT2440	Guaiacol	GGE and DDVA	Ca-lignosulfonate	Granja-Travez and Bugg, 2018
		*Pseudomonas fluorescens* Pf-5	Guaiacol	GGE and DDVA	Ca-lignosulfonate	Granja-Travez and Bugg, 2018
Monocopper polyphenol oxidase	Tfu1114	*Thermobifida fusca*	2,6-DMP	–	Alkaline lignin and sugarcane bagasse	Chen et al., 2016
Manganese-dependent superoxide dismutase	MnSOD	*Sphingobacterium* sp. T2	–	–	Organosolv and kraft lignin	Rashid et al., 2015

a *Lignin derived aromatic compounds or conventional peroxidase substrates*.

b *-, not examined in the paper*.

c *PAH is a polymeric compound, but not lignin*.

#### DyP-Type Peroxidases

DyP was first discovered in fungi as a unique heme-containing peroxidase, which is differentiated from plant peroxidase by the absence of a histidine residue involved in the heme-binding motif (Sugano et al., 2007). To date, more DyP-type peroxidases have been found in bacteria than fungi or other eukaryotes, and bacterial enzymes were shown to play a key role in lignin depolymerization, emphasizing its importance as bacterial peroxidase (Brown and Chang, 2014; Colpa et al., 2014). DyP-type peroxidase has been categorized into four subfamilies based on their amino acid sequence, A-D class, which is re-grouped as I, P, and V in a new classification considering structure based-sequence alignment (Yoshida and Sugano, 2015; Zamocky et al., 2015). Note that the A-C classes of DyP that are discussed in this review as DyPs in class D are primarily fungal enzymes.

DyPs in class A are reported to have a Tat-dependent secretion signal, which suggests they are involved in extracellular lignin depolymerization (Colpa et al., 2014). For example, a putative heme-containing DyP found from *B. sutilis* KCTC2023 (BsDyP) showed oxidative activity toward various substrates, including 2,2′-azino-bis(3-ethylbenzothiazoline-6-sulphonic acid) (ABTS) and Reactive Blue dye (Min et al., 2015). In addition to using an oxidation indicator, BsDyP also exhibited efficient decomposition of veratrylglycerol-β-guaiacyl ether (VGE), a dimeric lignin model compound containing the β-*O*-4 bond. At pH 3 and 50°C, conversion of VGE to veratryl aldehyde by BsDyP was observed in the presence of hydrogen peroxide, giving rise to a final conversion rate of 53.5% of within 2 h of reaction. DyP from *T. fusca* (TfuDyP) is also included in class A showing periplasmic export by Tat-system (van Bloois et al., 2010). The Fraaije group evaluated the catalytic potential of TfuDyP using different classes of substrate, including flavors, lignin-derived monomers, and dyes (Lončar et al., 2016). The oxidative activity of TfuDyP toward kraft lignin and guaiacylglycerol-β-guaiacyl ether (GGE), dimeric lignin model compound, was demonstrated (Rahmanpour et al., 2016). Especially, the formation of new products, generated from the dimerization of GGE, was identified by HPLC, although TfuDyP encouraged oxidative coupling rather than cleavage of the linkage. The thermophilic actinomycete, *Saccharomonospora viridis*, also contains DyP-type peroxidase (SviDyP) in subfamily A (Yu et al., 2014). When tested with an azo dye, SviDyP was active in wide pH (5–10) and temperature (50–80°C) ranges, showing the highest activity at pH 7 and 70°C. With these beneficial properties, SviDyP was applied to pulp bleaching, which enhanced the brightness and changed the morphology, resulting in it being considered as a promising candidate for further industrial applications. Additional A-type DyPs were reported in *Escherichia coli* and *Streptomyces* (Sturm et al., 2006; Liu et al., 2011; de Gonzalo et al., 2016).

Unlike the A subfamily, DyPs in the B (as well as the C) class do not have disclosed signal peptides for secretion, inferring that they are in the cytoplasmic phase of the cell (Colpa et al., 2014). Although the possibility of export has been proposed with the assistance of encapsulin, which is co-transcribed with *dypB*, in *Rhodococcus* strains, it still remains unclear (Tamura et al., 2015; Putri et al., 2017). Nevertheless, B-type peroxidase has been prominently studied owing to its versatile and sensitive activity. Ahmad et al. (2011) identified significant reduction in lignin deconstruction by a *R. jostii* RHA1 mutant carrying *dypB* deletion in comparison to the wild type strain, which emphasizes the role of DyPB in lignin breakdown. Based on the finding, characterization of the RjDyPB mutant having N246A was conducted with several lignin-related substrates including nitrated lignin, kraft lignin, and GGE as β-aryl ether compound, showing high oxidation activity in the colorimetric assay. In addition to the colorimetric assay, HPLC and GC-MS analyses showed the release of syringaldehyde as a deconstructed product from kraft lignin. Furthermore, its capacity was significantly enhanced to provide a 5.4–23-fold increase in the presence of MnCl_2_ (Singh et al., 2013). *P. fluorescens* Pf-5 was found to contain three DyP-type peroxidases, i.e., two DyPB and one DyPA (Rahmanpour and Bugg, 2015). Among these three enzymes, the best action was reported with DyP1B, demonstrating activity against all the substrates that were used. In particular, the oxidation of Mn(II) and kraft lignin was observed only by DyP1B. Moreover, in the treatment of wheat straw lignocellulose with DyP1B, the release of a lignin fragment was identified by HPLC. MS data suggested that the fragment was consistent with a compound containing two-aryl C3 units, which were a G and H units, respectively. Another important B-type DyP was found in *P. putida* MET94. In Santos's report, PpDyP exhibited higher activities and a wider scope of substrates, including phenolic and non-phenolic lignin units, compared with BsDyP (Santos et al., 2014). Later, Marins's group conducted an extensive study on PpDyP to characterize its integrated redox and catalytic properties and the construction of mutants for increased activity (Mendes et al., 2015; Brissos et al., 2017). The directed evolution of PpDyP generated the 6E10 variant carrying three mutations, E188K, A142V, and H125Y. This mutant showed noticeably improved efficiency in the oxidation of a lignin-related source, such as syringyl-type phenolics, GGE and kraft lignin, and displayed 100-fold higher catalytic efficiency toward 2,6-dimethoxyphenol (DMP) (Brissos et al., 2017). Another meaningful feature in this mutation is that this variant was found to preferably react under alkaline conditions, pH 8.5, whereas the wild-type enzyme and typical B-type DyP prefer an acidic (pH 4–5) environment. Recently, it has been reported that Asp-143 and Arg-232 residues of B-type DyP from *K. pneumoniaea* (KpDyP) and *E. lignolyticus* (ElDyP) play an important role in the decomposition of hydrogen peroxide by the enzyme structure analysis (Shrestha et al., 2017; Pfanzagl et al., 2018). Analysis of enzymes based on the structure will be helpful in rational design of enzymes for lignin depolymerization.

A limited number of enzymes have been characterized for DyP in the C subgroup, of which representatives are known from the genomes of proteobacteria, actinobacteria and cyanobacteria (Zamocky et al., 2015). Nevertheless, a strong peroxidase, DyP2, was identified within *Amycolatopsis* sp. 75iv (Brown et al., 2012). The activity of DyP2 was determined with ABTS, two dyes, and a manganese cation. Here, DyP2 also showed high activity as a Mn-peroxidase, which was comparable with that of fungal Mn-peroxidase. The breakdown of GGE also occurred in the presence of DyP2 and hydrogen peroxide, proving its capability of lignin depolymerization. The effect of DyP2 on lignin was also verified by heterologous expression within the *P. putida* strain. The overexpression and secretion of DyP2 within *P. putida* A514 showed ~2-fold higher CFU than the wild-type strain when they were cultivated on lignin as the sole carbon source, suggesting DyP2 was involved in the depolymerization of the lignin substrate (Lin et al., 2016). In 2009, Ogola et al. (2009) published their study of a cyanobacterium mobilizing C-type peroxidase, which is in the *Anabaena* sp. strain PCC7120. This heme-dependent peroxidase, AnaPX, showed substantial activity on phenolic compounds, such as guaiacol and pyrogallol, and this was significantly increased 50-fold by the redox mediator syringaldehyde. Recently, another bacterial DyP within the C class was discovered from *Streptomyces avermitilis*, following AnaPX and DyP2 (Sugawara et al., 2017). Even though SaDyP2 was found to be active on a typical oxidation indicator, further research would be required to determine the actual activity of lignin modification.

#### Laccase and Laccase-Like Multicopper Oxidase (LMCO)

The use of laccase, multicopper-containing oxidase, has been proposed for a wide range of bio-industrial uses, including bioremediation, lignin modification, and bio-bleaching of pulp and even biofuel cells (Arias et al., 2003; Beneyton et al., 2011; Zheng et al., 2012; Chandra and Chowdhary, 2015; Wang et al., 2015). In fact, fewer types of bacterial laccase are known and the efficiency in their wild type is not as high as that of the fungal enzyme. However, laccases from bacterial species have some advantages over those from fungi, such as thermo- and halo-tolerance, resulting in their consideration as suitable catalyst for industrial applications (de Gonzalo et al., 2016).

As a representative of lignin depolymerizing bacteria, *Streptomyces* species have been known to possess laccase (Fernandes et al., 2014). Based on the discovery of laccase within the strain, some strategies have been suggested to produce laccase at high yield within the culture. One strategy suggested by Niladevi et al. (2008) was to use a laccase inducer. By examining several aromatic compounds, pyrogallol was chosen as the best aromatic inducer to produce laccase from *S. psammoticu* at the level of 116 U/g, which was an ~2-fold increase compared to the control. The use of 1 mM of pyrogallol, at the optimal condition, was able to enhance the production yield up to 3.9-fold higher level. Within the culture of two *Streptomyces* strains, *S. lavendulae* and *S. cinnamomensis*, the optimization of the medium formula was conducted for the simultaneous production of laccase and lignin peroxidase. The use of an optimal formulation was able to enhance the production and control the ratio of laccase:LiP activity as well (Jing, 2010; Jing and Wang, 2012).

Similar to the studies of DyP, in many studies laccase was characterized not only by an oxidation indicator, such as ABTS and a variety of dyes, which suggested their potential as lignin modification enzymes, but also by lignin-related compounds or lignin to show their capability of lignin depolymerization directly. From a genomic analysis of *Pantoea ananatis* Sd-1, four putative laccase were identified, designated as Lac1-Lac4. Among them, only Lac4 was found to be able to oxidize phenolic compounds at acidic pH 2.5 (Shi et al., 2015). The ability of Lac 4 to decompose lignin was also evaluated by an *in vitro* assay using lignin. With the addition of ABTS as a mediator, 38% of lignin was observed to undergo degradation, and GC-MS analysis revealed the generation of LMW aromatic compounds, including 1,4-benzenedicarboxaldehyde, benzenepropanoic acid and phenol, which are considered to be the basic phenolic units possibly found in natural lignin. Laccase from *S. ipomoea* was also characterized with lignin-related compounds, such as *p*-coumaric acid, ferulic acid, and kraft or organosolv lignin prepared from softwood and spruce (Moya et al., 2011). In this study, the polymerization of lignin and lignan was also observed to show the reactivity of this laccase. Small laccase (SLac) is differentiated from typical laccase by containing only two domains for Cu binding rather than three, resulting in a smaller size (Machczynski et al., 2004). Majumdar reported four SLac from *S. doelicolor* A3(2), *S. lividans* TK24, *S. viridosporus* T7A, and *Amycolatopsis* 75iv2 (Majumdar et al., 2014). Initially, a mutant strain deficient of the SLac encoding gene was used to investigate its role in lignin decomposition. The generation of APPL only in the wild-type strain confirmed that SLac governs the lignin process. Then, all four enzymes were assessed for their activity toward lignin by using lignin model compounds with a β-aryl ether structure and ethanosolve lignin. Analysis of the product showed that one compound was identified as vanillin at A_340_, providing evidence for the laccase-related lignin modification.

Several examples of laccase-like multicopper oxidase (LMCO) have been reported within the bacterial strains. In 2001, a multicopper oxidase, CueO, was found in *Escherichia coli*, which confers tolerance to copper-induced damage (Grass and Rensing, 2001). Then, its oxidative activity was investigated using 2,6-DMP, a typical substrate for laccase, and polycyclic aromatic hydrocarbon (PAH), but the activity toward the lignin-related compound was not determined (Grass and Rensing, 2001; Zeng et al., 2011). Instead, CueO from *Ochrobactrum* sp. showed activity toward β-aryl ether and biphenyl lignin as model compounds of lignin (Granja-Travez et al., 2018). In the assay of oxidizing lignosulfonate, the generation of vanillin was observed as lignin-deconstructed fragment. Structural analysis showed similarity with CueO from *Escherichia coli*. Another LMCO that is well-studied is CotA from *Bacillus* strains. CotA, which is the protein originally related with the outer spore coating, was found to have similarities with multicopper oxidase, especially in terms of structural features, suggesting that CotA is a laccase (Hullo et al., 2001). Based thereupon, the oxidation of CotA from *B. subtilis* was evaluated with syringaldazine and several dyes to demonstrate its laccase activity (Hullo et al., 2001; Wang et al., 2016). Sufficient activity was observed at pH 7.2, even exhibiting high tolerance to organic solvents, including acetone, ethyl acetate, and chloroform. Then, *B. subtilis* was also examined with 45 aromatic chemicals, including 13 aromatic carboxylic acids and aromatic aldehydes, which are all lignin-related compounds, and displayed considerably high activity surpassing that of enzymes from *Streptomyces* (Ihssen et al., 2015). CotA from *B. licheniformis* and *B. pumilus* was also demonstrated to be an enzyme suitable for lignin modification via the oxidation of lignin-derived phenolics, such as guaiacol and syringic acid (Koschorreck et al., 2008; Ihssen et al., 2017). In the case of a *B. pumilus* CotA, mutant carrying L386W/G417L/G57F (abbreviated as WLF) was constructed by site-directed mutagenesis to enhance the catalytic efficiency, and a method was established for the efficient expression of WLF within *Escherichia coli*, which renders it a suitable candidate for industrial applications (Chen et al., 2017; Luo et al., 2018).

Recently, multicopper oxidase was identified in *Pseudomonas* genus. CopA from *P. stutzeri* was found by functional screening using a biosensor developed to respond to monomeric phenolics (Strachan et al., 2014). Further evaluation showed that industrially purified lignin incubated with CopA in the presence of Cu(II) released newly formed compounds, including 2,6-dimethoxybenzen-1,4-diol and 4-hydroxy-3methoxybenzoic acid, detected by GC-MS. *P. putida* KT2440 and *P. fluorescens* Pf-5 have been elucidate to retain CopA-type multicopper oxidase (Granja-Travez and Bugg, 2018). These researchers conducted comprehensive studies to determine the CopA involvement in lignin oxidation. They oxidized the lignin model compounds, GGE and 5,5′-dehydrodivanillate (DDVA) to obtain the dimerized compound. The reaction of lignosulfonate led to the production of vanillic acid. In addition, *P. putida* KT2440 mutated by deleting *copA* genes showed reduced growth on aromatic compounds. All these results suggested that CopA is able to act as laccase.

A different type of oxidase was found in *T. fusca* and *Sphingobacterium* sp. T2, showing activity toward lignin modification similar to that of laccase. Chen et al. (2016) reported a monocopper polyphenol oxidase identified within *T. fusca*. In addition to an oxidation test with 2,6-DMP, oxidative activity was determined with alkaline lignin and bagasse. The treatment of alkaline lignin with this oxidase led to a decrease in the total phenolic content, as confirmed by spectral scanning and size exclusion chromatography analysis. Moreover, modification of the lignin structure in bagasse was observed by LC-MS. The other is a bacterial manganese-dependent superoxide dismutase within *Sphingobacterium* sp. T2, which was found in proteomic analysis (Rashid et al., 2015). Two enzymes, MnSOD1 and MnSOD2, were both shown to be active toward organosolv and kraft lignin as well as the lignin model compound. The reaction generated several oxidized products, some of which were found to be formed by the cleavage of aryl-C_α_ and C_α_-C_β_ linkange such as dihydroxybenzoic acid, 5-hydroxyvanillic acid, and 2-methoxyhydroquinone.

## From Low-Molecular-Weight Lignin Product to Bioproducts

### Catabolic Pathways for Aromatic Compounds Within Bacteria

**Figure 1:**
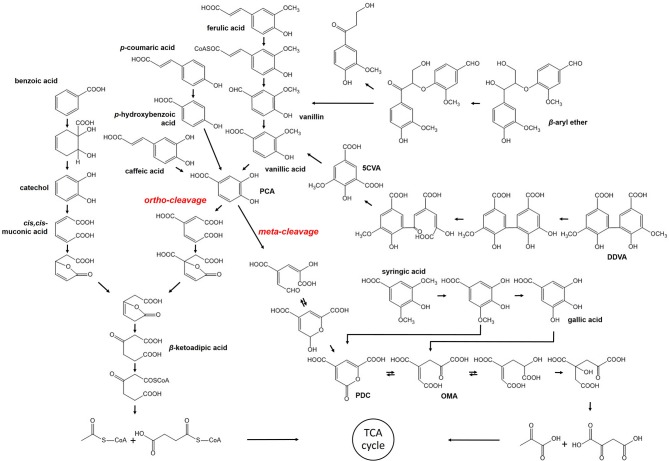
Bacterial pathway for degradation of lignin-derived compounds.

Most of the bacterial strains mentioned in the previous section have also been found capable of degrading lignin-derived oligomers or aromatic monomers, except for cellulolytic thermophiles. These strains converted high-molecular-weight polymers to compounds of lower molecular weight, followed by uptake to provide a source of energy. Several pathways within bacteria, in which LMW or monomeric compounds are digested via stepwise reaction to produce the important intermediate, such as protocatechuate (PCA) and catechol, have been discussed, even though they are not yet completely understood (Figure 1).

β-aryl ether such as GGE is a dimeric compound that contains the β-O-4 linkage, which is dominantly found in the lignin structure up to ~50% (Masai et al., 1999; Zakzeski et al., 2010). Therefore, cleavage of the β-aryl ether is significant in the degradation of lignin-derived LMW compounds and observed in several lignolytic strains, including *Sphingomonas paucimobilis, Delftia acidovorans, Rhodococcus equi*, and so on (Masai et al., 2007). The metabolism has been well-characterized in *S. paucimobilis* SYK-6 (Masai et al., 2007). The β-aryl ether is catabolized to vanillic acid by NAD-dependent dehydrogenase, LigD, and glutathione-dependent β-esterase, LigEFG, which is coupled with the reduction of glutathione (Gall et al., 2014a,b). After oxidation of the compound, ether cleavage takes place as a stepwise reaction. In the first step, LigE or LigF are involved in the reduction of glutathione by nucleophilic attack, but their different chirality preferences lead them to attack the opposite enantiomers (Masai et al., 2003). Additionally, LigG was found to have no esterase activity. Nevertheless, LigG is essential in this cleavage reaction as it is solely able to remove glutathione from β-thioether intermediate. The product formed by β-esterase is then metabolized to vanillic acid, which is further degraded by another pathway described below.

The biphenyl structure is also included in polymeric lignin. Fortunately, the mineralization of biphenyl compounds has been extensively studied within bacterial strains capable of degrading PCBs (Pieper, 2005). The conversion of DDVA, a model of the different types of biphenyl, was also reported to take place via a catabolic pathway within *S. paucimobilis* SYK-6 (Peng et al., 2002). This reaction is initiated by *O*-demethylation with LigX, and followed by ring opening via meta-cleavage of dioxygenase, LigZ. Finally, the two units are split, producing 5-carboxyvanillate. Subsequently, a decarboxylation reaction occurs to convert 5-carboxyvanilliate to vanillic acid, which is catalyzed by LigW. Peng et al. (2005) reported the identification of LigW2 as the second decarboxylase. Demonstration of the growth of *S. paucimobilis* SYK-6 on DDVA led to the finding that *ligW2* contributes to the conversion of 5-carboxyvanilliate as much as *ligW*. In addition to *S. paucimobilis*, the DDVA catabolic pathway was found in *P. putida, P. fluorescens, S. viridosporus*, and several soil bacteria (Crawford et al., 1981).

The degradation of lignin-derived monomeric compounds is known to occur via two different pathways and they are well-explained with *S. paucimobilis* SYK-6 and *P. putida* KT2440, respectively. In the case of ferulic acid, a precursor for lignin synthesis, the catalyzing enzymes are identical. Feruloyl-CoA synthetase starts the conversion of ferulic acid, encoded by *ferA* in *S. paucimobilis* and *fcs* in *P. putida* (Jiménez et al., 2002; Masai et al., 2007). Then, feruloyl-CoA is converted to vanillin with the help of FerB of *S. paucimobilis* or Ech of *P. putida*, which is further converted to vanillic acid via vanillin dehydrogenase. The feruloyl-CoA synthetase also catalyzes the transformation of *p*-coumaric acid and caffeic acid, but the following reactions produced *p*-hydroxybenzoic acid and PCA, respectively. The important intermediate along the degradation pathway is PCA, converged from most of the lignin-derived phenolics discussed in this section, including the conversion of vanillin produced by the cleavage of the dimeric compound. However, PCA proceeds through the different cleavage reactions within two strains. First, in *S. paucimobilis* SYK-6, PCA is converted by meta-cleavage instigated by 4, 5-dioxygenase, LigAB, and further degraded by LigCIJK to finally generate oxaloacetate and pyruvate (Masai et al., 2007). In contrast, ortho-cleavage occurs during the conversion of PCA to muconate within *P. putida* KT2440, which is catalyzed by the 3,4-dioxygenase, PcaGH (Jiménez et al., 2002). According to Bugg's report, more bacteria use the ortho-cleavage pathway (Bugg et al., 2011). *P. putida* KT2440 provides acetyl-CoA and succinyl-CoA as the final product via β-ketoadipate in which gene products from *pcaBCDIJF* are involved. Therefore, the pathway for aromatic compound catabolism in *P. putida* KT2440 is referred to as the upper β-ketoadipate pathway, which is observed in many other soil bacteria as well, such as *Acinetobacter* sp. ADP1, *Burkholderia* sp., and some other *Pseudomonas* strains (Jiménez et al., 2004; Fischer et al., 2008). This pathway covers the degradation of benzoic acid in another branch via catechol, the downstream degradation of which also forms β-ketoadipate.

### Conversion of Lignin to Biochemicals

Various soil bacteria are able to degrade aromatic compounds via the β-ketoadipate pathway and produce renewable chemicals (Table 3). Within this pathway, several intermediates that are practically useful can be found, including vanillin, which is widely used in the food industry as flavor additive (da Silva et al., 2009). Sainsbury's report suggested the production of vanillin from lignin by engineered *R. jostii* RHA1 (Sainsbury et al., 2013). This strain was constructed by deleting vanillin dehydrogenase, catalyzing the conversion of vanillin to vanillic acid, to block the downstream reaction and accumulate vanillin. When the mutant was cultivated with the minimal media containing both glucose and wheat straw biomass, vanillin was produced with 96 mg/L of titer. The pathway within *R. jostii* RHA1 was also manipulated to generate the aromatic carboxylic acid, which could be a source for the production of bio-plastic (Mycroft et al., 2015). PCA is normally cleaved by 3,4-dioxygenase (ortho-cleavage) and converted to a muconic acid compound (Jiménez et al., 2002). However, the conversion was re-routed to a meta-cleavage by insertion of 2,3-dioxygenase from *Paenibacillus* sp. JJ-1b or 4,5-dioxygenase from *S. paucimobilis*, which finally gave rise to pyridine 2,5-dicarboxylic acid (2,5-PDCA) and 2,4-dicarboxylic acid (2,4-PDCA), respectively. One percent wheat straw lignocellulose within the culture was transformed to generate maximally 125 mg/L of PDCA. Re-routing the cleavage of PCA and catechol introducedinto *P. putida* KT2440 enabled the production of pyruvate to be accelerated (Johnson and Beckham, 2015). In this case, catechol was catabolized by the 2,3-meta-cleavage to replace succinate with pyruvate as the product. Furthermore, PCA cleavage by 4,5-dioxygenase from *S. paucimobilis* served to generate two molecules of pyruvate. Finally, two substrates, p-coumaric acid and benzoic acid, which are degraded via PCA and catechol, respectively, were utilized by this engineered strain, and converted to pyruvate to increase the yield 5-fold. Integration of the lactate dehydrogenase A encoding gene from *B. Taurus*, finally led to the production of L-lactate from pyruvate to obtain a yield of 41.1%.

**Table 3:** Lignin-based chemical production.

	**Strains**	**Genotype**	**Substrates**	**Titers**	**References**
Vanillin	*Rhodococcus jostii* RHA045	*R. jostii* RHA1 derivative, *Δvdh*	Wheat straw cellulose	96 mg/L	Sainsbury et al., 2013
	*Pseudomonas putida* KT2440	*P. putida* KT2440 derivative, *Δupp Δ*PP_0166-0168 *Δvdh Δ*PP_3827-3832 ΔPP_2680 ΔPP_0545 ΔPP_1948 Ptac::*ech*:fcs	Ferulic acid	1.3 g/L	Graf and Altenbuchner, 2014
Lipid	*Rhodococcus opacus* DSM1069	Wild type	O_2_-treated kraft lignin	67 mg/L	Wei et al., 2015
			Ultrasonicated EOL	4 mg/g^a^	Kosa and Ragauskas, 2013
	*Rhodococcus opacus* PD630 and *Rhodococcus jostii* RHA VanA-	Wild type	Alkali-extracted lignin (from corn stover)	330 mg/g^b^	He et al., 2017
	*Rhodococcus opacus* PD630	Wild type	Laccase-treated kraft lignin	145 mg/L	Zhao et al., 2016
PHA	*Pseudomonas putida* KT2440	Wild type	Alkaline pretreated liquor (APL)	252 mg/L	Linger et al., 2014
	*Pseudomonas putida* A_DVJ4C1_	*P. putida* A514 carrying pTDV and pGJ4C1	Biorefinery waste	161 mg/L	Lin et al., 2016
	*Pseudomonas putida* A_xyl_alkKphaGC1_	*P. putida* A514 carrying pTP_xylA_phaGC1	Vanillic acid	246 mg/L	Wang et al., 2018
	*Pandoraea* sp. ISTKB	Wild type	4-hydroxybenzoic acid	409 mg/L	Kumar et al., 2017
	*Cupriavidus basilensis* B-8	Wild type	Kraft lignin	128 mg/L	Shi et al., 2017
			Alkaline pretreated liquor (APL) (from Rice straw)	450 mg/L	Si et al., 2018
	*Ralstonia eutropha* H16	Wild type	4-hydroxybenzoic acid	435 mg/L	Tomizawa et al., 2014
Muconic acid	*Pseudomonas putida* KT2440-JD1	*P. putida* KT2440 derivative, randomly mutated on *catR*, R50C	Benzoic acid	n.m.	Van Duuren et al., 2011
	*Pseudomonas putida* KT2440-CJ103	*P. putida* KT2440 derivative, *ΔpcaHG*::Ptac:*aroY,ΔcatRBC*::Ptac:*catA*:*dmpKLMNOP*	*p*-coumaric acid	13.5 g/L	Vardon et al., 2015
			Base-catalyzed depolymerized (BCD) lignin liquor	0.5 g/L	Rodriguez et al., 2017
	*Pseudomonas putida* KT2440-CJ184	*P. putida* KT2440 derivative, *ΔpcaHG*::Ptac:*aroY*:*ecdB*:*ecdDΔcatRBC*::Ptac:*catA*	*p*-coumaric acid	15.6 g/L	Johnson et al., 2016
	*Pseudomonas putida* MA-9	*P. putida* KT2440 derivative, *ΔcatBC ΔendA-1 ΔendA-2* Pcat::*catA*:*catA2* Pgro-4::*dmpKLMNOP*	Hydrothermal depolymerized pine lignin	13 g/L	Kohlstedt et al., 2018
	*Amycolaptosis* sp ATCC 39116 MA-2	*Amycolaptosis* sp ATCC 39116 derivative	Guaiacol	3.1 g/L	Barton et al., 2018

a *mg lipid/g us-EOL*.

b *mg lipid/g CDW*.

*n.m., not mentioned in the paper*.

Polyhydroxyalkanoate (PHA) is another promising material applicable to the production of bioplastics in various ways. Therefore, the conversion of lignin to PHA has been regarded as a valuable reaction. Linger et al. (2014) evaluated the production of PHA by *P. putida* KT2440 by studying monomeric lignin model compounds and APL in which these compounds are possibly present. They confirmed that a model culture consisting of a heterogeneous mixture containing *p*-coumaric acid, ferulic acid, glucose, and acetic acid was able to assimilate all the substrates within 48 h, except for ferulic acid, and 34% of PHA was accumulated. Moreover, the use of actual APL as substrate led to the accumulation of 32% of PHA. In this study, mcl-PHA produced from APL was converted to hydrocarbon fuels, demonstrating the valorization of lignin. *P. putida* A514 was also reported to generate PHA from vanillic acid and kraft lignin (Lin et al., 2016; Wang et al., 2018). A combination of several strategies was employed within the strain to enhance the yield of mcl-PHA, which includes overexpression of heterologous DyP and vanillin dehydrogenase to facilitate the utilization of lignin and lignin-derived compounds (Lin et al., 2016). The final mutant strain was able to accumulate PHA using biorefinery waste as the sole substrate, achieving 160 mg/L under N-source limited conditions. When the transcription level was optimized within *P. putida* A514 by a strong promoter with its regulator protein induced by xylose, PHA production from vanillic acid reached 246 mg/L, which is the highest record among the reported titer (Wang et al., 2018). Apart from the aforementioned work, PHA production from lignin within other bacterial strains has also been investigated. For example, Tomizawa et al. (2014) screened a bacterial strain that has both the ability to accumulate PHA and to efficiently utilize the lignin-derived metabolite, which is *Ralstonia eutropha* H16. PHA production was determined within *Pandoraea* sp. ISTKB as well with the lignin model compounds and kraft lignin. The use of *p*-hydroxybenzoic acid as the substrate resulted in the highest PHA accumulation, which was consistent with the amount of biomass (Kumar et al., 2017). Moreover, genomic and proteomic analyses were implemented, and enabled the two important gene clusters involved in lignin degradation and PHA polymerization to be identified (Kumar et al., 2018). The production of PHA by *Cupriavidus basilensis* B-8 from kraft lignin and lignin fragmentation was demonstrated by SEM and GPC analyses (Shi et al., 2017).

The availability of *P. putida* KT2440 in the bio-production of value-added chemicals was one again demonstrated with the generation of muconic acid. The first study on the accumulation of cis, cis-muconic acid (MA) was published in 2011, where a mutant was generated by chemically induced mutation (Van Duuren et al., 2011). This variant strain, which was shown to grow on benzoate only in the presence of glucose, was able to produce MA from benzoate, which was derived from deficient CatR, the transcriptional regulator of the *cat* operon responsible for catechol degradation; in addition, the strain induced the expression of *catA2*, of which the gene product is the second catechol 1,2-dioxygenase, within the *ben* operon for the conversion of benzoate to catechol. Subsequently, an effort to produce MA was reported with the acceptance of a wide range of compounds (Vardon et al., 2015). In this report, the compound degraded via the PCA branch of the β-ketoadipate pathway was funneled to MA by eliminating PcaHG for the ring cleavage of PCA and by re-routing to catechol with insertion of the PCA-decarboxylate encoding gene. This engineered strain, CJ103, was used to accumulate MA from *p*-coumaric acid and depolymerized corn stover (Vardon et al., 2015; Rodriguez et al., 2017). Additionally, the MA synthesized within *P. putida* was purified and further converted to adipic acid and nylon by chemical hydrogenation and polymerization (Vardon et al., 2015; Kohlstedt et al., 2018). Recently, upgraded MA production was reported with a *P. putida* KT2440-derived mutant, where the co-expression of AroY and its cofactor EcdBD accelerated the conversion of PCA to catechol (Johnson et al., 2016). A different access method using the metabolically engineered *Amycolatopsis* sp. ATCC 39116 was reported to produce MA by the metabolism of guaiacol-based lignin monomer (Barton et al., 2018). Deletion of the genes producing putative muconate cycloisomerase led to the synthesis of as much as 3.1 g/L MA from guaiacol. MA was also obtained with this mutant from guaiacol-enriched lignin hydrolysate prepared by the hydrothermal conversion of pine, thereby demonstrating its potential to achieve valorization.

As a biofuel, lipids are in demand for bioconversion by employing a bacterial strain. The *Rhodococcus* strain harbors an aromatic compound catabolic pathway via β-ketoadipate and also shows preference for acetyl-CoA as a substrate for fatty acid synthesis, which can be derived via this catabolic pathway (Kosa and Ragauskas, 2012). Two oleaginous *Rhodoccocus, R. opacus* DSM 1069 and PD630, efficiently utilized a variety of lignin model compounds, pretreated lignin, and whole lignocellulosic biomass for lipid production (Kosa and Ragauskas, 2013; Wang et al., 2014; Wei et al., 2015). The co-fermentation of two strains, *R. opacus* PD630 and *R. jostii* RHA1 showed improved growth on lignin and lipid generation with a synergetic effect (He et al., 2017), as demonstrated for the enhanced depolymerization within the co-culture of *Citrobacter* strains (Chandra and Bharagava, 2013).

Lignin valorization to produce useful chemicals still has unlimited potential by the versatile pathways within bacteria. Several reports have described the chemical reactions for lignin-derived phenolic adhesive and resin, and the synthesis of polyurethane foam (Jin et al., 2010; Ramires et al., 2010; Pan and Saddler, 2013). The synthesis of divanillin-derived polyurethane by a chemical catalyst has also been reported, and the formation of divanillin with the bacterial enzyme, TfuDyP, has been identified (Lončar et al., 2016; Gang et al., 2017). The possible replacement of these chemical reactions by bacterial systems would enlarge the market for lignin conversion.

## Bacterial Biosensors for Lignin Transformation

As mentioned before, the bioconversion of lignin to other chemicals has been achieved by using various cells or mutant strains. Although enzymes and cells related to lignin valorization are widely distributed in nature, only a fraction of them have been studied thus far. In addition, the low lignin transformation activity of these enzymes and strains is a major limitation for the industrial application of lignin. Transcriptional regulator-based whole cell biosensors are one of the most powerful tools to screen strains or enzymes capable of efficient lignin conversion, and would be a good choice to overcome this limitation. These microbial cell-based biosensors have been applied to numerous fields, including environmental analysis and medical diagnosis (Gu et al., 2004; Lee et al., 2013; Gui et al., 2017).

**Figure 2:**
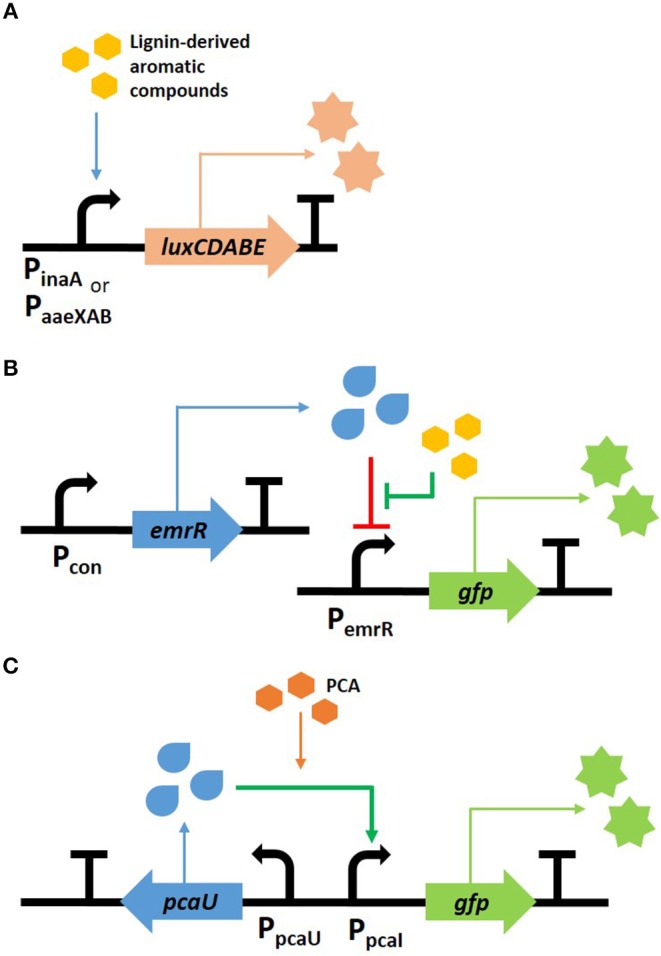
Scheme of biosensors for phenolic compound detection. Two *E. coli* sensors were developed to detect aromatic compounds using luminescent **(A)** and fluorescent **(B)** genes, and a fluorescent *P. putida* sensor was developed to respond to PCA **(C)**.

In recent years, bacterial biosensors used for the detection of lignin transformation were generally constructed by the fusion of fluorescence or luminescence reporters with promoters or transcription factors such that they were responsive to lignin-derived aromatic compounds (Figure 2). For example, two *Escherichia coli* biosensors were developed to exhibit a luminescent response to aromatic compounds. Here, the promoter regions of *inaA* and the *aaeXAB* operon encode the pH-inducible protein involved in stress responses and the *p*-hydroxybenzoic acid efflux system, respectively. These two regions, which were selected by transcriptional analysis, were fused with luciferase genes, *luxCDABE* (Lee et al., 2012). The luminescence increased when lignin-derived aromatic compounds, such as ferulic acid, *p*-coumaric acid, and vanillin, were added; however, sugar-derived furfural and HMF, which are also present within the pretreated lignocellulose, did not induce the luminescent signal (Figure 2A). These biosensors were used to determine the toxicity within pretreated lignocellulosic biomass with the aim of providing a non-inhibitory substrate for the biofuel production, as shown in the examples of uses (Lee and Mitchell, 2012; Monnappa et al., 2013). Another *E. coli* sensor was established by Hallam's group to detect mono-aromatic compounds resulting from lignin depolymerization. The sensing module “PemrR-GFP” was constructed by expressing GFP under the control of the EmrR promoter from the *emrRAB* multidrug efflux system operon (Figure 2B). This sensor was subsequently used to screen and identify bacterial strains containing the lignin transforming phenotype. The work ultimately led to the discovery of a novel multicopper oxidase within *P. stutzeri* (Strachan et al., 2014). Ho et al. (2017) modified this sensor, which enabled them to increase the sensitivity and selectivity of its fluorescent response by modeling analysis. Employing this sensor to screen the enzymes in a high-throughput system successfully screened 147 clones that were able to convert kraft lignin to vanillin and syringaldehyde, from 42,520 fosmid clones.

*Rhodococcus* and *Pseudomonas*, which are lignin transforming strains, were used to construct a bioreporter strain, thereby expanding their uses for lignin conversion (DeLorenzo et al., 2017; Jha et al., 2018). Several biosensors derived from *R. opacus* PD630 were developed to detect the intracellular nitrogen level or aromatic metabolites. In particular, a nitrogen sensor, which was constructed using the promoter region of LPD03031 gene encoding a putative nitrite extrusion protein, was able to provide useful information for lipid production from lignin. This is because *R. opacus* prefers low nitrogen conditions for increased TAG synthesis (DeLorenzo et al., 2017). Unlike the other sensors discussed here, which respond to various aromatic compounds, the *P. putida* KT2440 sensor is unique in that it only reacts toward protocatechuate (PCA), the central intermediate found in the catabolic pathway of lignin-derived aromatic compounds (Jha et al., 2018). The transcription factor PcaU from *A. baylyi* ADP1 was fused with superfolder GFP (sfGFP) as a reporter protein (Figure 2C), and evolved by modulating the protein-DNA interaction within the promoter and the protein-protein interaction of the dimer. The optimal sensor, indicated as PcaU 1.2, showed a dose-dependent response to PCA. The biosensor module was introduced into mutant cells with various genetic backgrounds and the intracellular accumulation of PCA from 4-hydroxybenzoic acid was evaluated. The strain showing the highest fluorescence was deficient of PcaHG, in which case the accumulated PCA cannot be further metabolized; furthermore the other strain from which PobA was deleted resulted in the reaction with the lowest yield because 4-hydroxybenzoic acid is not converted to PCA.

Biosensors have several benefits over physico-chemical analysis, such as a fast and sensitive response, simple preparation, and *in situ* detection, which makes these sensors useful tools in the field of lignin valorization. Accordingly, the development and utilization of additional diverse biosensors for lignin-derived aromatic compounds are foreseen.

## Challenges and Perspectives in Bacterial Lignin Valorization

Although lignin transformation in biorefinery has promised advanced process for the production of value-added chemicals as the alternative for a petroleum-based product, the structural complexity of lignin has made its utilization rate low. To enable the lignin valorization, the huge efforts have been made into each step, such as lignin characterization, biomass pretreatment, isolation of depolymerized compounds, and catalytic or thermochemical conversion, which are well-discussed in recent literature studies (Beckham et al., 2016; Cao et al., 2018; Ponnusamy et al., 2018; Wang et al., 2019).

In this review, we suggested that biological lignin valorization, in particular by bacterial microorganisms, could be a breakthrough for the efficient lignin utilization. As stated previously, bacteria show a wide spectrum of the aromatic substrate utilization or resistance, high adaptability in new environments, and easy genetic manipulation. Moreover, many researchers have discovered various lignolytic enzymes and catabolic pathway of aromatic compounds in bacterial cell, all which could facilitate the conversion of lignin to bio-chemicals in a single strain. The simultaneous lignin depolymerization and chemical production, called “consolidated bioprocessing,” has been proposed and the capability of bacterial strain has been proved (Linger et al., 2014). However, for the industrial feasibility of this bioprocessing of lignin, we still face several challenges needed to be solved, with largely two objectives: 1) efficient lignin depolymerization to supply considerable LMW lignin-related carbon substrate and 2) a significant increase in yield of value-added product from aromatic compounds.

Despite the discovery of various lignolytic enzymes, the enzyme research is still needed for the efficient extracellular decomposition of HMW to LMW lignin by bacterial strain. Especially, for the consolidated process, the enzyme is necessary to exhibit high catalytic efficiency in a mild condition, like as in the culture medium, although the most of the enzymes known are active in acidic condition (Janusz et al., 2017). Protein engineering will allow us to obtain improved lignin-decomposing enzyme. A variant of PpDyP could be an example, of which the activity toward kraft lignin at alkaline pH was enhanced by changing amino acids in the protein sequence (Brissos et al., 2017). Further discovery of novel efficient enzymes is also needed from the lignin-related environment with the aid of high-throughput sequencing and screening technology. Another issue on bacterial lignin depolymerization is that few secretion systems have been known. Several enzymes discussed in section Enzymes Involved in Lignin Depolymerization were reported as extracellular, but their secretion mechanisms involved were not fully studied, which makes it difficult to be engineered. Developing the enzyme secretion system in lignin-transforming strain will enable the active transport of enzyme, finally leading a considerable increase in lignin depolymerization.

For scaling up the lignin valorization, the high productivity of the target molecule should also be obtained by efficient uptake of substrate and their catabolism. Especially, as the high heterogeneity of lignin causes a diverse type of compounds in the depolymerized stream, the simultaneous uptake of those compounds is essential. Also, the lignin-depolymerized mixture possibly contains some compounds which are not preferred to consume by natural pathways, such as guaiacol (García-Hidalgo et al., 2019). In this sense, expanding the range of aromatic compounds as the substrate will be needed. Omics studies and synthetic biology will offer new genetic information and tools to identify and characterize the mechanism that can be further engineered. In addition, metabolic pathway could be tailored to funnel the substrate to the target product. Vardon et al. (2015) re-routed PCA to catechol pathway to maximize the production of cis, cis-muconate. Combining with computational studies would be more helpful for engineering, where metabolic flux could be possible to predict according to the design. Recently, the metabolic models of *P. putida* have been developed with single gene knockout by computational tool for the biochemical production (Tokic et al., 2019).

Here, we have several issues to be figured for significant lignin valorization. Nevertheless, several promising candidates have been suggested, including *Pseudomonas* species having DyP-type peroxidase (Salvachúa et al., 2015). In future work, engineering should be applied toward enzyme and bacterial strain to get an ideal bacterial system, where efficient lignin-degrading enzymes are sufficiently transported out and the aromatic compounds are efficiently assimilated, which are directly channeled into the product. Additionally, paired bacterial system or stepwise fermentation could also be possible for the efficient bacterial valorization.

## Conclusions

The valorization of lignin is essential to increase the sustainability and economic feasibility of the bio-refinery process, although the decomposition of lignin is complicated by its structural rigidity. Lignin valorization has been studied using catalytic, thermochemical, and biological approaches for many decades. Among biological approaches, few studies related to bacterial lignin decomposition were reported as compared with those involving fungi. However, various studies are currently underway because of its ease genetic manipulation and its ability to produce value-added chemicals using lignin-related aromatics. Decomposition of the lignin polymer was performed by DyP-type peroxidases and laccase secreted from bacteria. And LMW compounds from lignin was converted into value-added products. The results of lignin valorization by bacteria are still insignificant. However, new approaches using a bacterial biosensor, synthetic biology, and various “omic” studies are expected to lead to the discovery of a number of more effective lignolytic bacteria and enzymes that could be investigated for lignin utilization. These bacteria and enzymes could be potential candidates to achieve future lignin valorization.

## Author Contributions

All authors listed have made a substantial, direct and intellectual contribution to the work, and approved it for publication.

### Conflict of Interest Statement

The authors declare that the research was conducted in the absence of any commercial or financial relationships that could be construed as a potential conflict of interest.
